# Edaphic factors and plants influence denitrification in soils from a long-term arable experiment

**DOI:** 10.1038/s41598-020-72679-z

**Published:** 2020-09-29

**Authors:** Ian M. Clark, Qingling Fu, Maïder Abadie, Elizabeth R. Dixon, Aimeric Blaud, Penny R. Hirsch

**Affiliations:** 1 Sustainable Agriculture Sciences Department, Rothamsted Research, Harpenden, Hertfordshire AL5 2JQ UK; 2 College of Resources and Environment, Huazhong Agricultural University, Wuhan, 430070 Hubei People’s Republic of China; 3 Computational and Analytical Sciences Department, Rothamsted Research, North Wyke, Devon, EX20 2SB UK; 4 Present Address: School of Applied Sciences, Edinburgh Napier University, Sighthill Campus, Edinburgh, EH11 4BN UK

**Keywords:** Soil microbiology, Microbial ecology

## Abstract

Factors influencing production of greenhouse gases nitrous oxide (N_2_O) and nitrogen (N_2_) in arable soils include high nitrate, moisture and plants; we investigate how differences in the soil microbiome due to antecedent soil treatment additionally influence denitrification. Microbial communities, denitrification gene abundance and gas production in soils from tilled arable plots with contrasting fertilizer inputs (no N, mineral N, FYM) and regenerated woodland in the long-term Broadbalk field experiment were investigated. Soil was transferred to pots, kept bare or planted with wheat and after 6 weeks, transferred to sealed chambers with or without K^15^NO_3_ fertilizer for 4 days; N_2_O and N_2_ were measured daily. Concentrations of N_2_O were higher when fertilizer was added, lower in the presence of plants, whilst N_2_ increased over time and with plants. Prior soil treatment but not exposure to N-fertiliser or plants during the experiment influenced denitrification gene (*nirK, nirS, nosZ*I*, nosZ*II) relative abundance. Under our experimental conditions, denitrification generated mostly N_2_; N_2_O was around 2% of total gaseous N_2_ + N_2_O. Prior long-term soil management influenced the soil microbiome and abundance of denitrification genes. The production of N_2_O was driven by nitrate availability and N_2_ generation increased in the presence of plants.

## Introduction

Sustainable agriculture for an increasing global population requires a balance between optimizing fertilizer inputs and minimizing adverse outcomes whilst increasing food production. Emissions of the greenhouse gas nitrous oxide (N_2_O) from agricultural soils due to bacterial denitrification make a significant contribution to global warming^1^. When soils are wet and O_2_ availability is limited but there is sufficient organic matter, a common condition in temperate agricultural soils, nitrate (NO_3_^−^) can act as an alternative electron acceptor and is reduced to N_2_O by many different groups of facultatively anaerobic bacteria^2,3^. This occurs with different types of fertilizer, whether organic, biological (e.g. legumes), urea or inorganic ammonia/nitrate compounds^4^. Some bacteria can further reduce N_2_O to N_2_ which is not environmentally harmful but nevertheless reduces nitrogen use efficiency, wasting N fertilizer inputs. In a survey of genome-sequenced prokaryotes, 7% contained denitrification genes exemplified by the nitrite reductase gene *nirK,* the alternate gene *nirS* or the gene encoding nitrous oxide reductase, *nosZ*^5^. However, not all prokaryotes with a nitrite reductase gene carry *nosZ* and in others the gene is not active. Consequently, for these organisms, the final denitrification step is the release of N_2_O. Also, there are also a substantial number of bacteria that contain *nosZ* without *nirK* or *nirS* and are assumed to sequester and reduce N_2_O, emitting N_2_^6^. Many soil fungi can also denitrify: their contribution is minor when compared to that of bacteria in agricultural systems and arable soil but increases post-harvest and after incorporation of organic residues^7^. Fungi form only a small proportion of the soil microbiome under growing crops, and fungal denitrifiers contain *nirK* but not *nosZ* thus do not reduce any N_2_O they generate to N_2_^8^. N_2_O generated by other processes including nitrification is at relatively low levels compared to the activity of denitrifying bacteria in anoxic, fertilized agricultural soils^9,10^.

The alternate nitrous oxide reductase genes have been known for many years but the significance of carrying either *nirK* (encoding a Cu-containing enzyme) or *nirS* (encoding a cytochrome *cd*_*1*_) remains unclear^11^. The two forms can occur within different individual species of the same genus but (with rare exceptions) they are mutually exclusive^12^. Recent stable isotope probing experiments to identify denitrification genes in soil indicated that *nirK* was the most abundant and active, with greater sequence diversity than previously recognised^13^. The *nosZ* genes are also a diverse group and have been divided into two clades: *nosZ*I in the Proteobacteria; *nosZ*II in the Gemmatimodates, Bacteroidetes and Choroflexi^14^.

Whilst there have been many surveys of N_2_O fluxes from agricultural soils, some using intact or repacked soil cores under lab conditions, others made in the field, few have specifically investigated the role of crop plants or measured N_2_ emissions. Most are concerned with soil organic carbon (SOC), nitrogen and N-fertilizer forms and rates of application, soil moisture and temperature^1,15,16^. There is considerable information on the roles of these drivers but the effects of interaction between plant and soil factors including the microbiome on denitrification is less clear. A compilation of field data concluded that the presence of plants increased N_2_O emissions and also the ratio of N_2_:N_2_O^17^. Experiments in air-free systems with Argon or Helium replacing N_2_ have indicated that arable soils with young plants evolve more N_2_O and N_2_ than controls with no plants^7,18^. However, plants were also implicated in reduced soil moisture and less N fertilizer remaining in soil, leading to lower denitrification^19^. A review of 26 separate studies investigating cover crops in the field showed that 40% resulted in lower and 60% in higher emissions compared to control soils without cover crops^20^. Presence or absence of legumes, high soil N, rainfall and crop residues were all implicated in these differences. However, a more recent meta-analysis of 129 publications indicated that the presence of cover crops significantly reduced N_2_O emissions compared to bare soil^21^. In an experiment using soil cores with artificial root exudates, no N_2_O was detected in the control soil in contrast to that receiving exudates^22^ whereas a field experiment comparing bare soil (chemical fallow) with non-legume cover crops found no significant difference in N_2_O emissions^23^. These contradictory results concerning N_2_O and N_2_ emissions from agricultural soil in the presence or absence of plants reinforce the need for a deeper understanding of the processes involved.

In this paper, we report on a glasshouse experiment designed to measure production of N_2_O and N_2_ in soils taken from plots with contrasting long-term treatments which were anticipated to alter microbial communities with potential consequences for the relative abundance of denitrification genes. Furthermore, we aimed to examine the impact of growing plants and of applying KNO_3_ fertilizer on gas production. The soils were taken from the long-term Broadbalk Winter Wheat experiment at Rothamsted Research (UK), where different fertilizers and N-rates have been applied, resulting in soils with distinctly different edaphic properties. Using conditions designed to promote denitrification, soils from different long-term treatments, in pots with or without wheat plants, were treated with ^15^N-labelled fertilizer, control soils receiving no fertilizer and sealed in chambers with ambient air. The plants had been grown to the stem elongation stage where root exudation is at a maximum^24^ and the roots had colonised the pots so all soil was “rhizosphere”, for comparison with unplanted controls. The concentrations of N_2_ and N_2_O in these sealed chambers were measured daily over 4 days to provide a snapshot of denitrification activity in the rhizosphere and the control bulk soil; soil DNA and mRNA were extracted at this point to assess the abundance and activity of denitrification genes. From this we show the relative importance of antecedent soil treatments that influence edaphic factors including the soil microbiome, crop plants and N-fertilizer in promoting denitrification.

## Material and methods

### Soils

In the Broadbalk Experiment, running since 1843 at Rothamsted Research (UK), different fertilizers and N-rates have been applied consistently to winter wheat. In 1882, one section (previously referred to as “wilderness”) was taken out of cultivation and left to regenerate to woodland^25,26^. We chose plots that received farmyard manure (FYM) at 35 t ha^−1^, no N fertilizer (N0) or 288 kg N ha^−1^ annually (N6), together with the woodland soil (Wood). Table 1 shows the soil properties and annual fertilizer applications of the four arable treatments and the plot converted to woodland.

**Table 1:** Fertilizer applications and soil properties for Broadbalk plots.

Treatments	Fertilizer year^−1^		Soil properties^a^						
	N kg ha^−1^	Other	% SOC	% total N	% clay	% silt	% sand	Bulk density	pH^b^
N0	None	PKMg	0.93	0.10	24.6	57.1	18.3	1.2	8.2
N6	288	PKMg	1.2	0.13	33.4	39.2	27.4	1.2	7.1
FYM	246^c^	FYM	3.21	0.31	23.3	50.9	25.8	1.1	7.8
Woodland	None	None	6.23	0.48	29.0	52.0	15.0	0.9	7.7

N-fertilizer is ammonium nitrate, applied as a single dose in April; FYM is applied in autumn at 35 T ha^−1^.

^a^Andy Gregory & Chris Watts, personal communication (bulk density is expressed as g cm^−3^ oven-dried soil).

^b^pH in H_2_O.

^c^Mean annual N content in 35 T FYM ha^−1^ (Andy Macdonald, personal communication).

Broadbalk is not fully replicated since it was set up before the advent of modern statistical design, hence each plot to be sampled was subdivided into three equally sized pseudoreplicates, each 9.3 m × 6 m in the arable plots and 9.3 m × 4 m in the FYM plot. The woodland area (80 m × 15 m) was also subdivided into equally sized pseudoreplicates. Ten soil cores (top 20 cm) were collected and pooled from each area in May 2014, generating three replicate soil samples for each of the four plots. Soil was sieved to 4 mm and stored at 4 °C for 14 days before transferring to pots; weighed subsamples were dried overnight at 80 °C to estimate the soil dry weight (dw).

### Plants and pots

A total of 48 pots were set up: three soil replicates from each of the 4 plots, with four treatments (+ /− wheat; + /− N fertilizer). Each Ø 10 cm pot contained 350 ml vol. soil; 24 pots were planted with 4 pre-germinated wheat seeds (*Triticum aestivum* cv Cadenza) per pot, grown 6 weeks and given foliar feed if required (diagnosed by slight yellowing of leaves) and watered as normal with tap water. At 6 weeks post germination, wheat root exudation is at a maximum^24^; previous experiments showed wheat roots to fill the pots at this stage. The 24 unplanted pots were set up and kept in glasshouse alongside those with wheat in a randomised block design and were watered at the same time as the plants to maintain soil moisture. Soil bulk and particle density had been measured previously in the source soils^27^ and was used to calculate the pore space. This enabled subsequent manipulation of the water-filled pore space (wfps).

### Chambers

When plants reached stem elongation but pre-flowering (c. 6 weeks post germination), the soils were adjusted to 95–100% wfps to create the anaerobic conditions conducive to denitrification, by adding sterile deionised water (sd H_2_O), or a solution of K^15^NO_3_ (99% atom enrichment) in sd H_2_O as to give equivalent to 100 kg N ha^−1^ (i.e. 40 µg N g^−1^ dw soil) in each pot. Immediately after adding water or fertiliser, a 2 L polycarbonate bottle with the base removed was placed over each pot with a SubaSeal at the top, as shown in Supplementary Fig. S1, creating closed chambers containing air for the 4 day sampling period.

### Gas sampling and measurement of N_2_ and N_2_O

The first samples (day 0) were taken immediately after sealing the chambers following the addition of K^15^NO_3_ fertilizer or water. Subsequently, the chambers were sampled at 24 h intervals for the next 3 days (day 1, day 2, day 3, day 4), with two ambient air samples taken at each sampling time. A single 22.5 ml air sample was taken at each timepoint for N_2_O analysis; for ^15^N isotope ratio sampling, a second 12 ml air sample was taken from each treatment fertilized with K^15^NO_3_ to measure ^15^N_2_-N and ^15^N atom% in N_2_O. The total (chamber plus pore space) volume and the soil dry weight in each pot was used to calculate N_2_O-N and ^15^N_2_-N g^−1^ dw soil for each replicate.

The N_2_O was measured using gas chromatography with an electron capture detector and an automated sample injection system; a TG2 trace gas analyser (Europa Scientific, now Sercon, Crewe, UK) interfaced to a Sercon 20–22 isotope ratio mass spectrometer (IRMS) was used to measure ^15^N enrichment of N_2_. Solutions of 6 and 30 atom% ammonium sulphate ((NH_4_)_2_SO_4_) were prepared and used to generate 6 and 30 atom% N_2_O^28^ and used as reference and quality control standards. Atmospheric air, with natural abundance ^15^N (0.3663 atom%) was used as the reference for N_2_. The N_2_ concentration in each sample was calculated from the ratios of the intensity of ion beams at mass to charge ratios 28, 29 and 30, using the equations of Stevens and Laughlin^29,30^.

### DNA and RNA extraction and amplicon sequencing

After 4 days, the chambers were removed. Soil samples were processed within 5 min to preserve the integrity of the RNA and DNA. In pots with wheat, the roots had spread throughout the soil and soil adhered to the roots at sampling. This soil was shaken off and mixed, similarly for unplanted pots, soil was mixed, a subsample taken, sieved to 2 mm and frozen in liquid N_2_ for subsequent DNA and RNA extraction using the RNA PowerSoil^®^ isolation kit and RNA PowerSoil^®^ DNA Elution Accessory Kit (MO BIO Laboratories, Inc) following manufacturer’s instructions^31^. RNA samples were DNAse treated to remove DNA contamination using the DNase Max Kit (Qiagen, Manchester, UK), following the manufacturer’s protocol. Direct PCRs and gel electrophoresis were carried out on DNAse treated RNA to confirm all contaminating DNA had been removed. The quantity and quality of extracted DNA and DNAse-treated RNA were analysed by fluorometer Qubit^®^ 2.0 dsDNA and RNA BR Assay Kits and Nanodrop microvolume spectrophotometer (Thermo Fisher Scientific).

The bacterial and archaeal diversity was determined from the 12 samples taken at the time of field sampling by amplicon sequencing of 16S rRNA genes using the primers 515F/806R, sequenced on Illumina’s MiSeq platform and analysed to phylum (sub-phylum for Proteobacteria) level and to OTU (97% sequence identity) using the QIIME 1.8 pipeline. The method was described in detail previously^32^. The amplicon sequence data for this study have been deposited in the European Nucleotide Archive (ENA) at EMBL-EBI under accession number PRJEB36852 (https://www.ebi.ac.uk/ena/browser/view/PRJEB36852).

### Quantification of bacterial and denitrification gene abundance

The primers used to detect the 16S rRNA genes, the alternative genes for nitrite reductase (*nirK* and *nirS*) and the nitrous oxide reductase gene *nosZ* clades I and II are shown in supplementary Table S1. Quantitative-PCR (qPCR) amplifications were performed in 10 µl volumes containing 5 µl QuantiFast SYBR Green PCR Master Mix for DNA and QuantiFast SYBR Green RT-PCR Master Mix for RNA (Qiagen, Manchester, UK), 0.1 µl of each primer (1 µM), 0.1 µl of QuantiFast RT Mix for RT-qPCR, 2 µl of template DNA at 5 ng µl^−1^ or 2–4 µl of RNA at 10 ng µl^−1^ and nuclease-free water (Severn Biotech, Kidderminster, UK) up to 10 µl, using a CFX384 Touch™ Real-Time PCR Detection System (Bio-Rad, Hemel Hempstead, UK). The standards for each molecular target were obtained using a tenfold serial dilution of PCR products amplified from an environmental reference DNA and purified by gel extraction using the Wizard^®^ SV Gel and PCR Clean Up System (Promega, Southampton, UK) following the manufacturer’s instruction then quantified by fluorometer Qubit^®^ 2.0 dsDNA BR Assay Kit (Thermo Fisher Scientific). Standard curve template DNA and the negative/positive controls were amplified in triplicate. Amplification conditions for all qPCR assays consisted of an initial denaturation at 95 °C for 5 min followed by 40 (two step) cycles; 95 °C for 10 s and 60 °C for 30 s. The RT-qPCR program had an initial reverse-transcription step at 50 °C for 10 min. Each amplification was followed by melt curve analysis (60 °C to 95 °C, with incremental readings every 0.5 °C) to confirm the specificity of each assay. Efficiency of amplification for each primer set was > 82% with *r*^2^ ≥ 0.996 (Supplementary Table S2). Results are expressed as gene copies g^−1^ dw soil.

### Soil properties

At the end of the pot trial, samples from each pot were oven-dried as described above, to measure moisture and estimate the final wfps. Extracts (1:5 soil dw:2 M KCl, shaken for 2 h at 300 rpm, 20 °C) were analysed for nitrite (NO_2_^−^), nitrate (NO_3_^−^) and ammonia (NH_4_^+^) using a Skalar colorimetric continuous flow Analyzer. Results are given as μg g^−1^ dw soil.

### Statistical analyses

GenStat 17th Edition (VSN International Ltd, Hemel Hempstead, UK) was used to perform ANOVA with soil origin, sampling date (i.e. time of incubation), presence or absence of plant and addition or not of fertilizer as factors, to compare values obtained from soil and gas analyses and from qPCR estimations of gene and transcript copy numbers. To check that each set of measured values met the assumptions of ANOVA and were normally distributed, residuals were plotted. If they did not show normal distribution, data was log-transformed and again checked for normal distribution of residuals. Where ANOVA results were significantly different (*P* < 0.05), means were further tested using Tukey’s post-hoc method in the GenStat multiple comparison menu with 95% confidence. Where appropriate, standard errors of difference of means (s.e.d.) are indicated. When only two treatments were compared, Student’s t-test in the Excel data package was used. The statistics package PAST v. 3.16^33^ was used to analyse the relative abundance of 16S rRNA amplicons using: SIMPER to determine the percentage contribution of each phylum to each treatment; non-metric multidimensional scaling (NMDS) analysis at the OTUs level based on the Bray–Curtis similarity index; PERMANOVA to assess the significance of the NMDS plot and Spearman’s rank correlations of gene abundance, soil properties and gas emissions. Unless otherwise indicated, statistically significant differences are assumed to occur at *P* ≤ 0.05 and are referred to as “significant” throughout the text; results with no significant differences are referred to as NSD.

## Results

### Soils and their microbial communities

The soil properties shown in Table 1 indicate variation in soil texture across the Broadbalk field, with less clay in the N0 and FYM plots, situated on the north side of Broadbalk field compared to N6 and woodland towards the south side. The soil pH ranged from 7.1 to 8.2, lowest in the mineral-nitrogen fertilized soil N6 and highest in the N0 soil that received no N fertilizer. The bulk density of woodland soil is much lower and the % SOC much higher compared to other soils; the FYM soil has lower bulk density and higher % SOC than the other arable soils. The ratio of SOC:total N was approximately 10:1 in the arable soils and 13:1 in the woodland soil.

**Figure 1:**
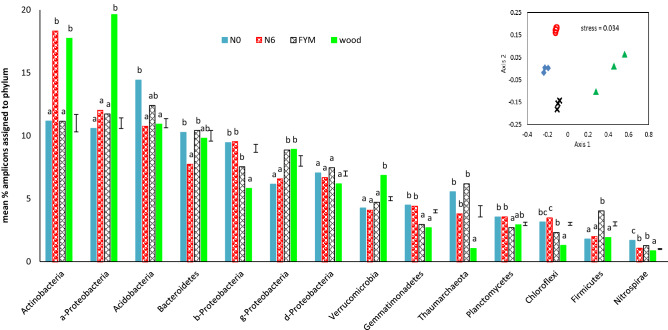
Relative mean abundance of prokaryotic phyla/sub-phyla in soils of origin on collection from the field. Phyla with at least 0.1% of the total community present in at least one soil treatment are included. Proteobacteria sub-phyla: a = alpha, b = beta, d = delta, g = gamma; s.e.d. for each group is shown; letters indicate mean significantly different means within each group (*P* = 0.05, according to Tukey’s post-hoc test on ANOVA). Insert top right shows NMDS plot of OTU for prokaryotic communities – PERMANOVA *F* = 9.477, *P* (same) = 0.0001.

The community structure of bacteria and archaea revealed by 16S rRNA amplicon sequencing of metagenomic DNA extracted from the soil samples, at collection from the field, shows significant differences, and distinct separation on a NMDS plot (Fig. 1). Of the 14 phyla (sub-phyla for the Proteobacteria) comprising > 0.1% of the community in at least one of the soils, only the δ-Proteobacteria did not show significantly different (*P* ≤ 0.05) mean abundance in at least one soil, according to ANOVA (Fig. 1). For example, the woodland soil has more α-Proteobacteria and Verrucomicrobia but fewer Thaumarchaeota (archaea) and β-Proteobacteria than the other treatments. Both the FYM and woodland soil have more γ-Proteobacteria and fewer Gemmatimonadetes; the FYM soil has more Firmicutes than the other soils (Fig. 1).

### 16S rRNA and denitrification gene abundance

**Figure 2:**
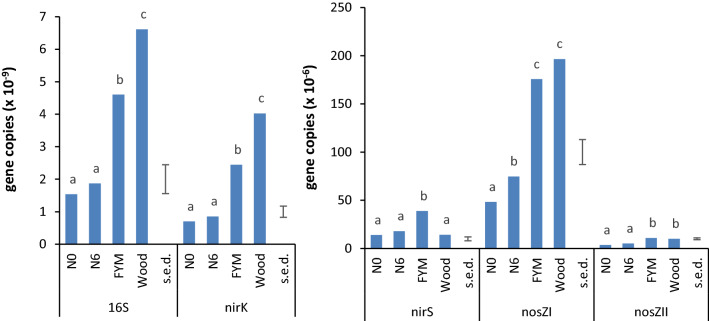
Gene abundance from qPCR at the end of the experiment, pooling all treatments for each soil of origin (n = 12); letters denote significantly different values within each set of genes (*P* = 0.05) according to Tukey’s post-hoc test in ANOVA; s.e.d. = standard errors of difference of means; note that 16S and *nirK* are plotted as 10^–9^, the other genes as 10^–6^ copies g^−1^ dw soil.

At the end of the experiment, DNA was extracted and amplified from all samples but sufficient RNA for further analysis was obtained only from the FYM and woodland soils which contained more organic matter and larger microbial communities. ANOVA comparing the abundance for each set of genes and transcripts measured using qPCR showed that the soil of origin had a significant influence in all cases (Table 2). However, other factors (presence/absence of wheat and addition or not of N-fertilizer) and interactions between them were not significant, except for *nosZ*I which was significantly influenced by the plant. Bacterial abundance indicated by 16S rRNA gene copy number was 2 × 10^9^ g^−1^ soil in the N0 and N6 soils and significantly higher in the FYM and woodland soil, 5 × 10^9^ and 7 × 10^9^ copies g^−1^, respectively (Fig. 2). This pattern of relative abundance was seen for *nirK* (7 × 10^8^–4 × 10^9^ copies), *nosZ*I (5 × 10^7^–2 × 10^8^ copies) and *nosZ*II (4 × 10^6^–1 × 10^7^ copies g^−1^ soil). The exception was *nirS* where N0, N6 and woodland soil had similar gene abundance (1 × 10^7^ copies g^−1^ soil) and FYM significantly more with 4 × 10^7^ copies g^−1^soil (Fig. 2). The ratio *nirK:nirS* gene copies in the woodland soil was 300:1, significantly more than the mean of 55:1 in the arable soils (*F*_3,32_ = 102.63, *P* < 0.001). Woodland also had a significantly higher ratio of *nir:nosZ* genes, 20:1 compared to 13:1 in the arable soils (*F*_3,32_ = 10.97, *P* < 0.001). This was influenced only by the origin of the soil, not the plant or fertilizer treatment (supplementary Fig. S2, Supplementary Table S3).

**Table 2:** ANOVA for soil edaphic factors and gene abundances.

	d.f.	Soil properties				Gene copy number from qPCR			
		wfps	NO_3_^–^N	NH_4_^+^-N	16S	*nirK*	*nirS*	*nosZ*I	*nosZ*II
Soil	*F*_3, 32_ *P*	9.58 < .001	12.8 < .001	46.75 < .001	61.0 < .001	124.6 < .001	47.66 < .001	145.4 < .001	25.5 < .001
Fertilizer	*F*_1, 32_ *P*	NS	321.5 < .001	NS	NS	NS	NS	NS	NS
Plant	*F*_1, 32_ *P*	17.05 < .001	444.4 < .001	25.69 < .001	NS	NS	NS	8.18 = 0.007	NS
Soil × fertilizer	*F*_3, 32_ *P*	NS	11.09 < .001	NS	NS	NS	NS	NS	NS
Soil × plant	*F*_3, 32_ *P*	9.09 < .001	21.91 < .001	NS	NS	NS	NS	7.56 < .001	NS
Fertilizer × plant	*F*_1,32_ *P*	NS	8.84 = 0.006	NS	NS	NS	NS	NS	NS
Soil × plant × fertilizer	*F*_3, 32_ *P*	NS	3.84 = 0.019	NS	NS	NS	NS	3.43 = 0.028	NS

The mean number of 16S rRNA transcripts overall in the woodland soil, 5.3 × 10^8^ copies g^−1^, was significantly higher (*t*_22_ = 2.23, *P* = 0.02) than the FYM soil with 1.8 × 10^8^ copies g^−1^. The same pattern was seen with *nirK* transcripts: 2.4 × 10^5^ copies in the woodland; 1.2 × 10^5^ copies in the FYM soil (*t*_22_ = 3.75, *P* < 0.001). There was no significant difference between the two soils for *nosZ*I transcripts which were much less abundant, mean 6 × 10^3^ copies g^−1^ soil.

### Soil properties at the end of the experiment

**Figure 3:**
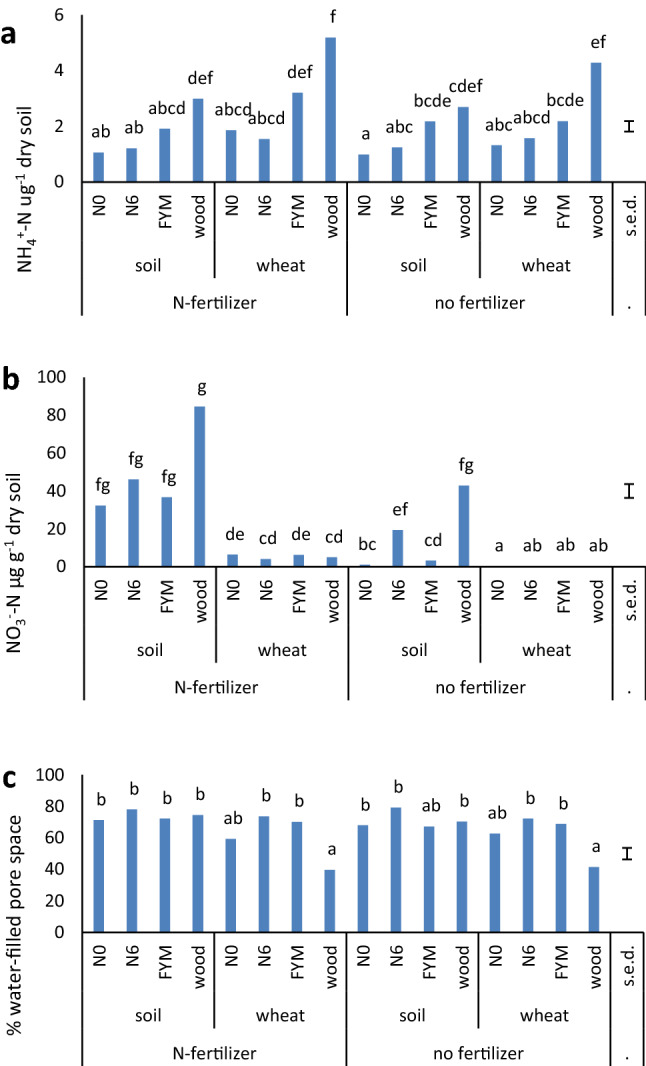
Soil properties at the end of the experiment. (**a**) concentration of NH_4_^+^-N; (**b**) NO_3_^–^-N; (**c**) % wfps; means for soils with all treatments (n = 3); different letters denote significantly different values according to Tukey’s post-hoc test in ANOVA (*P* = 0.05), s.e.d. = standard errors of difference of means for all samples.

The concentration of soil NO_2_^−^-N at the end of the experiment was below the limit of detection in most samples and is not included. The NH_4_^+^-N followed the same trend as the % N and bacterial abundance, significantly higher in the woodland soil (Fig. 3a). According to ANOVA, it was influenced by the presence of plants but not K^15^NO_3_-fertilizer additions (Table 2). This was confirmed using *t-*tests: the mean NH_4_^+^-N concentration for all soils with plants was 2.6 μg g^−1^ soil, significantly higher (*t*_46_ = 2.6, *P* = 0.007) than 1.6 μg g^−1^ for bare soils. The NH_4_^+^-N is around tenfold less than the NO_3_^−^-N in unfertilized soils, indicating nitrifier activity in the aerobic soils prior to setting up the chambers whereby soil pore saturation to create anaerobic conditions is predicted to reduce nitrification rates.

ANOVA showed the NO_3_^–^-N concentration to vary significantly between soils and to be influenced by both K^15^NO_3_ fertilizer additions and the presence of plants (Table 2). The NO_3_^–^-N concentration remaining in soils receiving no K^15^NO_3_ was significantly higher for woodland and N6 than for the N0 and FYM soils (Fig. 3b) and the overall mean in bare soil was 16.6 μg g^−1^, significantly higher (*t*_22_ = 3.0, *P* = 0.006) than in the presence of plants (0.36 μg g^−1^ soil). Where K^15^NO_3_ was applied, differences due to soil of origin was not significant (Fig. 3b) but the mean value for unplanted soils was 49.9 μg g^−1^, significantly higher (*t*_22_ = 6.0, *P* > 0.001) than where plants were present (5.4 μg g^−1^).

The % water-filled pore space (wfps), set at an estimated 95% at the start of the experiment, had fallen to 60–80% in most soils by the end, and to 40% for the woodland soils with wheat (Fig. 3c, Table 2). Water had drained from the pot into the tray and had also been redistributed around the sides of the chambers as condensation; plants but not K^15^NO_3_ fertilizer addition had a significant influence (Table 2). The overall mean wfps in all bare soil soils 72.6% was significantly higher (*t*_46_ = 3.3, *P* < 0.001) than 61.0% for all planted soils.

### Gas production

**Figure 4:**
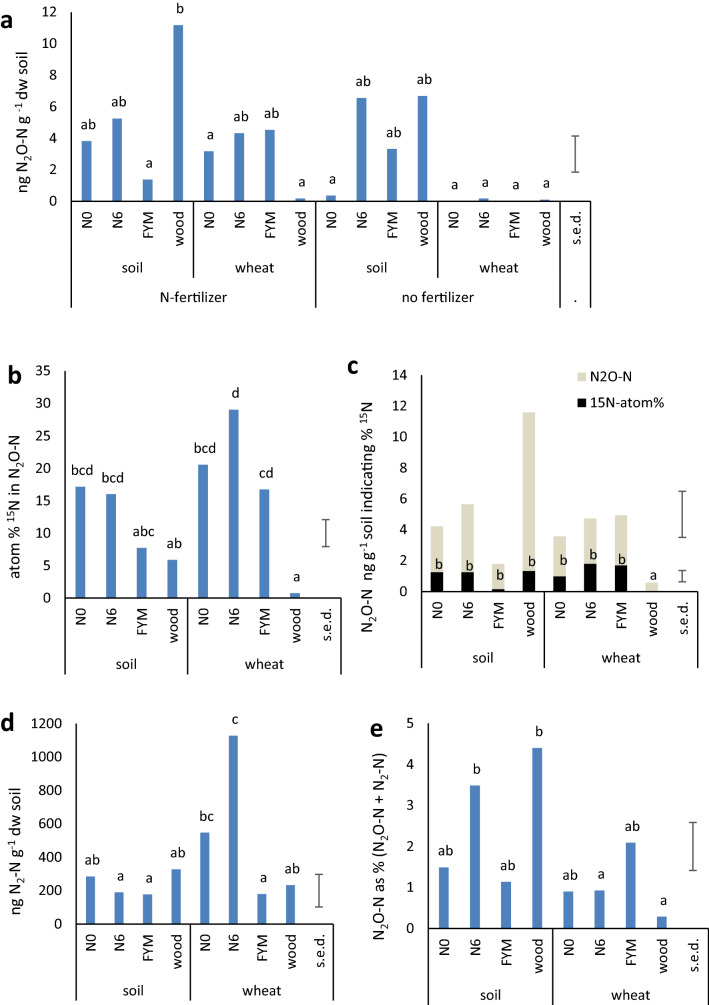
Mean gas production over 4 days. (**a**) N_2_O-N, all treatments (48 pots); (**b**–**e**) K^15^NO_3_-fertilized treatments only (24 pots). (**b**) ^15^N atom % measured in N_2_O; (**c**) N_2_O-N indicating %^15^N (upper s.e.d. relates to N_2_O-N; lower bar relates to ^15^N atom %), (**d**) N_2_-N, (**e**) N_2_O-N as % (N_2_-N + N_2_O-N). Different letters denote significantly different values according to Tukey’s post-hoc test in ANOVA (*P* = 0.05), s.e.d. = standard errors of difference of means for all samples.

Gas measurements made immediately after adding K^15^NO_3_ fertilizer or water and sealing the chambers (day 0) were similar to ambient values and were not included in subsequent analyses (e.g. mean N_2_O-N from 48 chambers was 0.27 ppm, s.e.d. 0.0035; ambient N_2_O in 10 glasshouse air samples was 0.28 ppm, s.e.d. 0.0048). Subsequent samples were taken at 24 h intervals (day 1–4) until the experiment concluded, the chambers were dismantled, and the soil was sampled. ANOVA indicated that the presence/absence of plants and K^15^NO_3_ had a significant effect on N_2_O-N but not the sampling date either alone or in combination with the other factors; in contrast, CO_2_ levels were additionally influenced by sampling date (Table 3). For this reason, each day was treated as a repeat sampling for N_2_O (Fig. 4a). In unfertilized soil, mean N_2_O-N from unplanted soil was 5.2 ng g^−1^, significantly higher (*t*_22_ = 3.0, *P* = 0.003) than with plants (1.1 ng g^−1^ soil). However, where K^15^NO_3_ was applied, the mean N_2_O-N was NSD in bare and planted soil. Over all treatments, the N_2_O-N measurements were highly variable with NSD between most means and overall differences due to the soil of origin were also NSD (Table 3.) The exception was significantly higher N_2_O-N in woodland compared to FYM soil where K^15^NO_3_ was applied and no plants were growing (Fig. 4a).

**Table 3:** ANOVA for gaseous losses from all soils and treatments.

ANOVAs	d.f.	N_2_O	CO_2_
All soils			
Date	*F*_3,128_ *P*	NS	11.26 < .001
Soil	*F*_3,128_ *P*	2.46, NS = 0.065	109.15 < .001
Fertilizer	*F*_1, 128_ *P*	22.33 < .001	9.24 = 0.003
Plant	*F*_1, 128_ *P*	39.9 < .001	61.94 < .001
Soil × fertilizer	*F*_3,128_ *P*	3.43 = 0.019	NS
Soil × plant	*F*_3, 128_ *P*	9.04 < .001	20.35 < .001
Fertilizer × plant	*F*_1, 128_ *P*	12.68 < .001	NS
Soil × fertilizer × plant	*F*_3, 128_ *P*	NS	NS

The mean CO_2_ measured was 1 µg g^−1^ dw soil for all soils whether plants were growing or not, with or without K^15^NO_3_ application. The exception was bare woodland soil, with a mean of 25 mg g^−1^ dw soil (Supplementary Fig. S3). This indicates a similar rate of production and consumption by soil and plants for soils taken from the long term arable treatments, with only the woodland soil with high SOC and microbial biomass producing significantly more CO_2_ than the system could consume, peaking at 48 h (Supplementary Fig. S4).

Measurement of ^15^N was only possible in the K^15^NO_3_-fertilized plots. The proportion present in N_2_O, indicated by the ^15^N atom% (Fig. 4b), varied significantly between soils of origin. The sampling date and presence/absence of plants did not have a significant influence according to ANOVA (Table 4) but the mean value for all times and samples in bare soil, 11.7% was significantly (*t*_94_ = 2.0, *P* = 0.02) less than the mean value where plants were present (16.8%). The data was used together with N_2_O-N measurements to show that the proportion of ^15^N in N_2_O-N was significantly less from the woodland soil when wheat was present (Fig. 4c). This could be because the more open pore structure of the woodland soil (demonstrated by the drop in wfps at the end of the experiment) resulted in better root growth and proportionally greater uptake of the ^15^N-labelled fertilizer by the wheat.

**Table 4:** ANOVA for gas measurements in K^15^NO_3_-fertilized soils only.

	d.f.	N_2_-N	Total N_2_-N + N_2_O-N	N_2_O-N as % total N	d.f.	^15^N atom%	^15^N_2_O-N
Date	F_3,57_ *P*	4.15 = 0.01	4.1 = 0.011	2.96 = 0.04	F_3,64_	NS	NS
Soil	F_3,57_ *P*	8.13 < .001	8.24 < .001	NS	F_3,64_ *P*	33.26 < .001	10.7 < .001
Plant	F_1, 57_ *P*	15.62 < .001	14.83 < .001	15.89 < .001	F_1, 64_	NS	NS
Soil × plant	F_3, 57_ *P*	10.24 < .001	10.23 < .001	4.56 = 0.006	F_3, 64_ *P*	10.67 < .001	9.1 < .001

The N_2_-N measured in the K^15^NO_3_-fertilized plots showed that the date of sampling as well as soil of origin and plant had significant influences (Table 4). The presence of plants appeared to increase N_2_ production over time (supplementary Fig. 5), in contrast with total N_2_O-N production which showed no significant changes. The overall mean for all soils and times with plants was 533 ng g^−1^ N_2_-N, significantly more (*t*_47_ = 2.8, *P* = 0.004) than without plants (239 ng g^−1^ N_2_-N). In the absence of plants there was NSD between the mean N_2_-N in the different soils but when plants were present, N6 produced significantly more N_2_ than the FYM and woodland soils (Fig. 4d). The total gaseous N (^15^N_2_-N + N_2_O-N) was dominated by N_2_-N with an overall mean of 390 ng g^−1^ soil compared to 4 ng g^−1^ for N_2_O-N. However, the relative abundance showed a significantly higher % N_2_O-N in bare N6 and woodland soils compared to those with wheat (Fig. 4e) and the presence of plants had an overall significant effect according to ANOVA (Table 4).

### Relating edaphic and microbiological factors with gas production

To investigate factors influencing gaseous emissions, Spearman’s rank correlation was derived for gas, soil and microbial parameters for all 48 pots where N_2_O was measured (supplementary Table S4A) and the 24 pots where K^15^NO_3_ fertilizer was added and ^15^N_2_-N and ^15^N atom% in N_2_O-N was measured (Supplementary Table S4B). Mean values of gas concentrations from all four sampling times were used. Overall, N_2_O-N was moderately correlated to wfps and strongly correlated to NO_3_^–^-N, factors both known to support denitrification. However, in the fertilized soils the NO_3_^–^-N relationship was not apparent, presumably because the relatively high NO_3_^–^-N was not limiting. The N_2_O-N from fertilized treatments was positively correlated moderately with N_2_-N and highly with the ^15^N atom% in N_2_O-N. N_2_O-N as a % of (N_2_-N + N_2_O-N) and ^15^N atom% showed moderate negative correlation with NH_4_^+^-N (Supplementary Table S4b). There was no correlation between either N_2_O-N or N_2_-N and the total bacterial community indicated by 16S rRNA gene numbers, or with any of the denitrification genes although there was a moderate negative correlation between N_2_O-N and the *nir:nos* ratio in the subset of K^15^NO_3_-fertilized soils. Abundance of all the denitrification genes was strongly correlated with 16S rRNA indicating the relationship between SOC and microbial abundance, except for *nirS*. This relationship was supported further by a strong positive correlation of the 16S rRNA and denitrification genes with NH_4_^+^-N, derived from the mineralization of soil organic matter. There was no significant correlation between *nirS* and *nirK* although both correlated with *nosZ*I and *nosZ*II abundance. The ratio *nirK*:*nirS* showed weak negative correlation with the soil wfps, strong positive correlation with NH_4_^+^-N and moderate correlation to CO_2_ (positive) and, in the ^15^N-fertilized pots, ^15^N atom% (negative). The *nir:nos* ratio showed similar trends and additionally showed moderate negative correlation to N_2_O-N and, in the ^15^N-fertilized fertilized pots, to N_2_O-N as a % of (N_2_-N + N_2_O-N). Neither ratio showed significant correlation with N_2_-N. There were no statistically significant correlations between *nosZI* and *nirK* mRNA abundance and gaseous emissions.

The CO_2_ emissions (prominent only in woodland soil without plants) correlated with NO_3_^−^-N; 16S rRNA, *nirK* and *nosZI* gene abundance; and with N_2_-N in the K^15^NO_3_ fertilized subset*.*

## Discussion

### The role of microbial diversity and abundance

Microbial diversity was influenced by previous long-term treatment of the different plots, resulting in the distinctly different microbiome composition revealed by amplicon sequencing. These contrasting treatments: arable management with N-fertilizer (N6); no N (N0); organic FYM; and the woodland soil left untilled and unamended, provide very different scenarios and each appears to favour divergent combinations of phyla and subphyla as shown in Fig. 1. The overall size of the microbial biomass (also related to SOC) indicated by 16S rRNA gene abundance was related to the abundance of the denitrification genes *nirK* and *nosZ*I*,* which did not appear to be increased by the regular application of N fertilizer. The denitrification genes are relatively common across different phyla, and it is likely that although the community composition varied, gene abundance was not limiting in these experiments. In contrast to the production of CO_2_, N_2_ and N_2_O did not show statistically significant correlation to 16S rRNA gene abundance, nor to any of the denitrification genes although there was a moderate negative correlation between *nir:nos* ratio and N_2_O-N, N_2_O%, and ^15^N atom%. This indicated that as the proportion of nitrous oxide reductase genes (*nosZ*I + *nosZ*II) increased compared to nitrite reductase genes (*nirK* + *nirS*), there was proportionally less N_2_O in the chambers as it was converted to N_2_.

The biogeography of denitrifying bacteria and their genes, and differences in their relative abundances^22,34^, is one reason for the lack of consensus between different studies which attempt to define the most important genes. Edaphic factors such as pH are known to influence soil microorganisms: changing soil pH in different plots caused significant differences in denitrification gene abundance^35^. The relative abundance of *nirK* and *nirS* reported elsewhere appears to depend on soil properties and management: relatively more copies of *nirS* were reported in unfertilized bare fallow compared to nitrate or FYM-fertilized soil, where *nirK* was more abundant^36^. Our results, from different soils and treatments, are not directly comparable with these but are consistent with previous measurements in Broadbalk soil^25^. The relatively higher abundance of *nosZ*I compared to *nosZ*II is consistent with reports that *nosZ*I occurs in Proteobacteria^14^ which together form the most abundant phyla in all Broadbalk soils. Recently, changes in the diversity and abundance of *nirK* and *nosZ* genes expressed in soil microcosms have been related to N_2_ and N_2_O emissions^37^. In our experiments, we could measure transcription of *nirK* and *nosZ*I only in FYM and woodland soil and there were twice the number of copies of *nosZ*I per *nirK* in FYM compared to woodland soil which could explain the lower N_2_O and N_2_O:N_2_ measured in the FYM compared to woodland soil in the absence of plants although correlations were not significant. When plants are present, differences in the uptake of the added ^15^N fertilizer that limit the amount left in soil as a denitrification substrate may change this relationship (see below). We were surprised that 16SrRNA gene abundance was not found to be significantly lower in bare soil compared to planted pots containing rhizosphere soil. Bacterial cell numbers are higher in the rhizosphere than bulk soil^38^ but the qPCR assays in our study were probably insufficiently sensitive to detect this, compared to the much larger differences due to the source of the soil.

### The importance of plants in gas production

In water-saturated conditions conducive to denitrification, both nitrate fertilizer and plants had a major influence on N_2_O production. This agrees with the findings of a recent meta-analysis where fertilizer quantity and the presence of crops were the most important drivers influencing N_2_O emissions^21^. The relative abundance of the various genes involved in NO_2_^−^ and N_2_O reduction did not appear to drive the gasses measured in our experiments, indicating that denitrification was not constrained by abundance of *nirK, nirS, nosZ*I and *nosZ*II in the arable soils although differences in relative abundances (*nirK:nirS; nir:nosZ*) might explain the significantly greater N_2_O production in bare, fertilized woodland compared to FYM soil. The relatively low N_2_O-N measured in woodland soils where wheat was grown could reflect the drop to 40% wfps, conditions less conducive to denitrification. The proportion of ^15^N in N_2_O-N in bare woodland soil was lower than in the arable soils, and significantly lower where wheat was growing, indicating that some of the “extra” N_2_O in woodland soil arose from NO_3_^−^resulting from nitrification of NH_4_^+^ derived from the larger total N pool. The NH_4_^+^-N is likely to be a product of SOM mineralisation rather than a legacy of previous fertilization regimes; the results indicate that plants can stimulate the soil microbiome to increase this mineralisation. Nitrifying bacteria, archaea and fungi in soil can oxidize NH_4_ to NO_2_^−^ and subsequently NO_3_^−^, especially in aerobic conditions.

In our experiments, the most N_2_O was produced when fertilizer was added to bare soil, and least in unfertilized soil with wheat, and the concentration measured in the chambers did not change significantly over the sampling period. In contrast, N_2_ increased over time, with more produced when plants were present. The ^15^N atom% in N_2_O was also higher in soil with plants. The increase in N_2_ indicates that N_2_O was being actively reduced during the experiment, that production and consumption of N_2_O was in equilibrium, and full denitrification from NO_3_^–^ to N_2_ was more efficient in the presence of plants despite their competition for NO_3_^–^-N. Most microbes are more active in the presence of plants, stimulated by the energy-rich root exudates, and this may explain both increased ^15^N atom% in N_2_O and increased reduction of N_2_O to N_2_. In addition, this high degree of conversion of N_2_O to N_2_ may arise from the closed experimental system causing denitrifying microorganisms to remain in contact with N_2_O for longer than in open soil in the field, where N_2_O is likely to be rapidly lost to the atmosphere. In contrast to other experiments measuring the effect of small plants in enclosed chambers^7,18^, we used mature plants resulting in all soil being in contact with roots, effectively rhizosphere soil, for comparison to bulk soil in unplanted pots. This would have enhanced the plant effect, explaining the much higher conversion of N_2_O to N_2_ that we observed together with the reduction in soil NO_3_^−^-N which was rapidly assimilated by plants. The abundance of *nosZ* is reported to be influenced by resource availability, with relatively fewer copies detected in nutrient-poor environments and an increased proportion present in rhizosphere communities^34^. The relevance of other differences such as the presence of more copies of *nirS* in FYM soil, are unclear since they did not result in significant differences in gaseous emissions, although it should be acknowledged that the gene abundance was measured only at the end of the experiment and could have varied over the preceding 4 days.

### Soil factors driving denitrification

Emissions of N_2_O and N_2_ are known to fluctuate and much greater replication is needed to demonstrate which factors apart from NO_3_^−^ and wfps are driving denitrification. The closed design of our experiment with daily gas sampling did not allow measurement of gas fluxes but result indicate that the peak concentration of N_2_O was reached by 24 h and did not increase significantly after this time because it was being reduced to N_2_, which in contrast, increased each day in most treatments. Our observations that most N_2_O produced in soil is further reduced to N_2_ by active denitrifying bacteria is consistent with many reports. However, less N_2_ than N_2_O was measured in planted soil fertilized with KNO_3_ in a sealed system with He^7^ although the converse was observed in an earlier experiment with Ar^18^; fertilizer type and water saturation also influenced denitrification in these studies. Our system, in contrast, was designed to have an enhanced rhizosphere effect and growth of plants in a normal atmosphere. The maximum amount of K^15^NO_3_-N converted to N_2_ over the 4 days of our experiment was 4%, in the N6 soil, where 1.6 µg ^15^N_2_-N g^−1^ dw soil was detected after 4 days, derived from 40 µg K^15^NO_3_-N g^−1^ dw soil added when the chambers were sealed. With plants, ~ 5 µg NO_3_^−^-N g^−1^ soil remained after 4 days, suggesting that much more was taken up by the wheat than was available for denitrification. In other studies, N_2_O-N comprised 7% of (N_2_O-N + N_2_-N) emitted from cores of wet agricultural soil^39^ and 25% in saturated soils amended with artificial root exudates^40^. There are several reports that the ratio of N_2_O:N_2_ increases with the concentration of NO_3_^−^ in soil^7,29,41^ but the concentration of K^15^NO_3_ in our experiments, 40 μg N g dw soil^−1^ equivalent to 100 kg N ha^−1^, was relatively modest (and decreased by 85% over 4 days in the presence of plants) compared with many other studies in vitro*.* For example, the in vitro study^40^ with model root exudate applied 100 μg N g dw soil^−1^ and application rates cited in field surveys were 160–300 kg N ha^−1^ year^−1^^29^ and 200–500 kg N ha^−1^ year^−1^^41^, respectively. In our pots, NO_3_^−^ was more evenly distributed throughout the soil than in field applications, so avoiding localised high concentration activity hotspots. High concentrations of NO_3_^−^ are reported to inhibit nitrous oxide reductase and hence the reduction of N_2_O to N_2_^42,43^. In soil microcosms amended with with 50 μg NO_3_^–^-N g dw soil^−1^, N_2_O comprised > 1% (N_2_O + N_2_) after 48 h whereas adding 500 μg NO_3_^–^-N g dw soil^−1^ diminished N_2_ emissions by 38–90%^42^. Another study^44^ found fungal denitrification dominated initially, after high levels of straw and nitrate (200 mg NO_3_^–^-N g^−1^) were added to soil but when NO_3_^–^-N fell to 40 mg g^−1^ soil, N_2_ evolution dominated.

The constraints on measuring de novo N_2_ production in the presence of 78% N_2_ in air, make it difficult to design experiments without adding isotopically labelled N fertilizer and plants reportedly do not flourish when N_2_ in air is replaced by Ar or He. The acetylene inhibition method previously used to prevent conversion of N_2_O to N_2_ has many disadvantages^45^. Our experiment was designed to determine the effect of well-developed wheat plants at peak root exudation on denitrification, and on the bacterial genes involved, following saturation of the soil, rather than following the development of the plant and denitrification rates over time. We were constrained by the need to sample gas from the chambers manually: future experiments using repeat robotic gas sampling would offer great advantages. Over the short 4 days enclosure, we observed that plants decreased soil NO_3_^−^ and moisture levels and increased CO_2_, all of which may influence effect denitrification and could be investigated further.

It would be interesting to attribute the denitrification genes that we detected to the organisms from which they originated. Further studies to sequence amplicons may reveal whether different groups are present and (with improved mRNA extraction) active in the different soils, coupled with estimates of gene abundance over time (within 24 h and over days), and improved monitoring of gaseous emissions may help to explain the high variability observed. Nevertheless, our work reported here confirms that N-substrate (nitrate) availability is the main driver for N_2_O production in water-saturated soils and furthermore, it shows that the presence of plants promotes further reduction of N_2_O to N_2_, which is the major denitrification product. The relative abundance of the various genes implicated in denitrification did not play a major role in these experiments although long-term pre-treatment of soils had generated significant differences in the composition of their soil microbiomes.

## Supplementary information

Supplementary Information 1.

## Supplementary information

is available for this paper at 10.1038/s41598-020-72679-z. The amplicon sequence data for this study have been deposited in the European Nucleotide Archive (ENA) at EMBL-EBI under accession number PRJEB36852 (https://www.ebi.ac.uk/ena/browser/view/PRJEB36852).
