# Supplementary material for: Edaphic factors and plants influence denitrification in soils from a long-term arable experiment
Source: Sci Rep. 2020 Sep 29;10:16053. doi: 10.1038/s41598-020-72679-z (PMC7524710; doi:10.1038/s41598-020-72679-z)
Supplement: Supplementary file 1 — Supplementary Information 1. [file 41598_2020_72679_MOESM1_ESM.pdf]

Supplementary Information

Edaphic factors and plants influence denitrification in soils from a long-term arable experiment

Ian M. Clark[a]; Qingling Fu[a,b]; Maïder Abadie[a]; Elizabeth R. Dixon[c]; Aimeric Blaud[a,d]; Penny R. Hirsch[a]*

[a] Sustainable Agriculture Sciences Department, Rothamsted Research, Harpenden, Hertfordshire AL5 2JQ, UK

[b] College of Resources and Environment, Huazhong Agricultural University, Wuhan 430070, Hubei Province, PR China

[c] Computational and Analytical Sciences Department, Rothamsted Research, North Wyke, Devon, EX20 2SB, UK

[d] Current address: School of Applied Sciences, Edinburgh Napier University, Sighthill Campus, Edinburgh, EH11 4BN, UK

*corresponding author

penny.hirsch@rothamsted.ac.uk

tel: 0044 (0)1582 938669

fax: 0044 (0)1582 760981

Supplementary Information

Supplementary Fig. S1. Plant pots set up for gas collection: gas samples collected though SubaSeals.

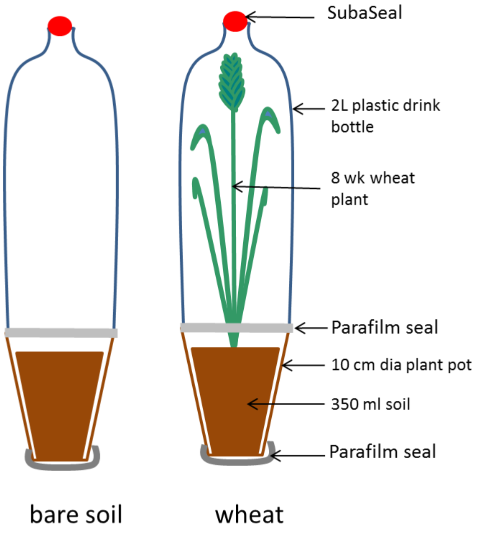

Supplementary Fig. S2. Ratio of nitrite reductase genes *nirK:nirS* (left) and nitrate reductase: nitrous oxide reductase genes (*nirS* + *nirK*) : (*nosZ*I + *nosZ*II)

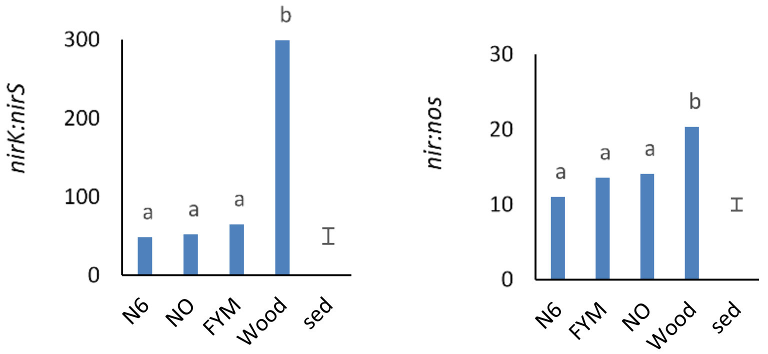

Supplementary Fig. S3. CO_2 production (mean measured over 4 days) with ambient CO_2 subtracted (hence negative values for some treatments).

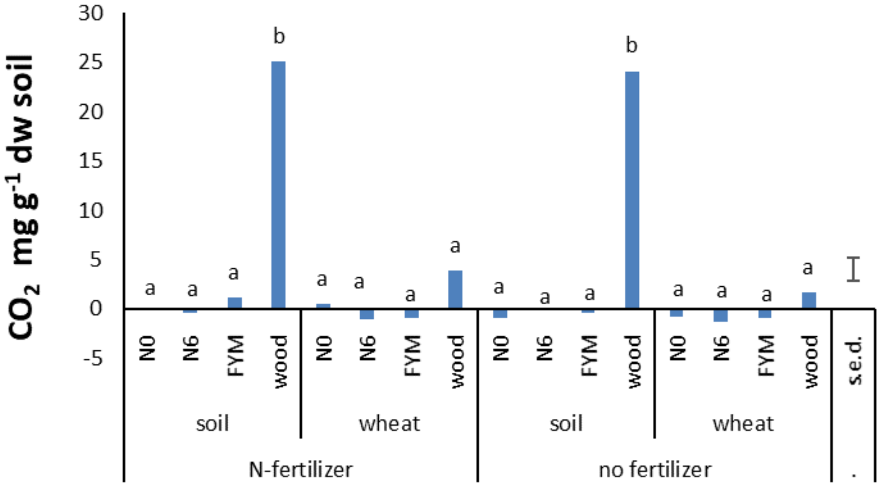

Supplementary Fig S4. CO_2 measurements made 24 h after the chambers were sealed (d1) and at 24 h intervals subsequently (d2, d3, d4).

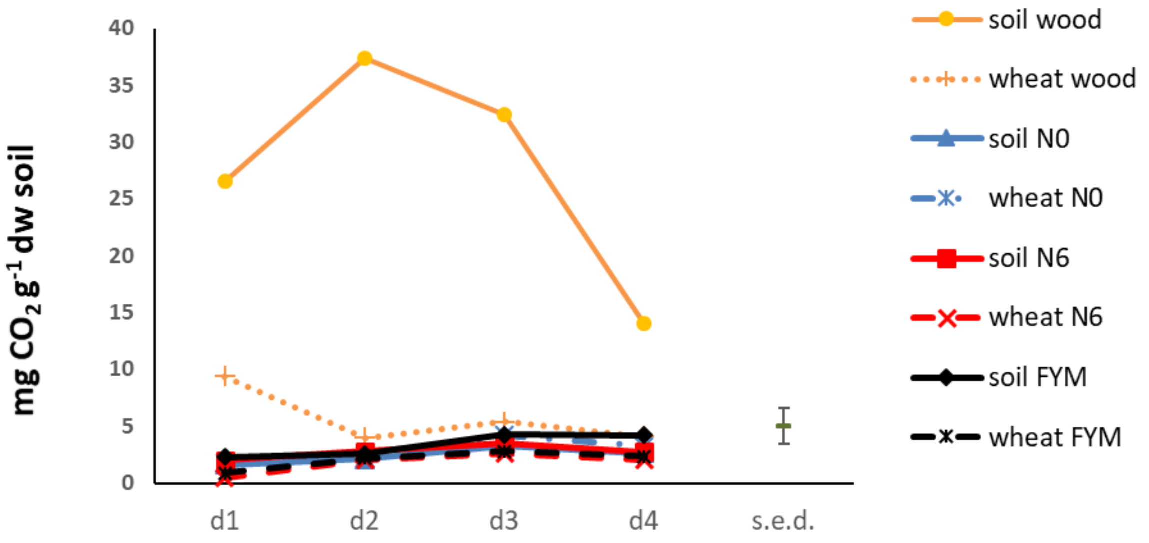

Supplementary Fig S5. N_2-N measurements made 24 h after $K^{15}NO_3$ fertilizer application (d1) and at 24 h subsequently (d2, d3, d4).

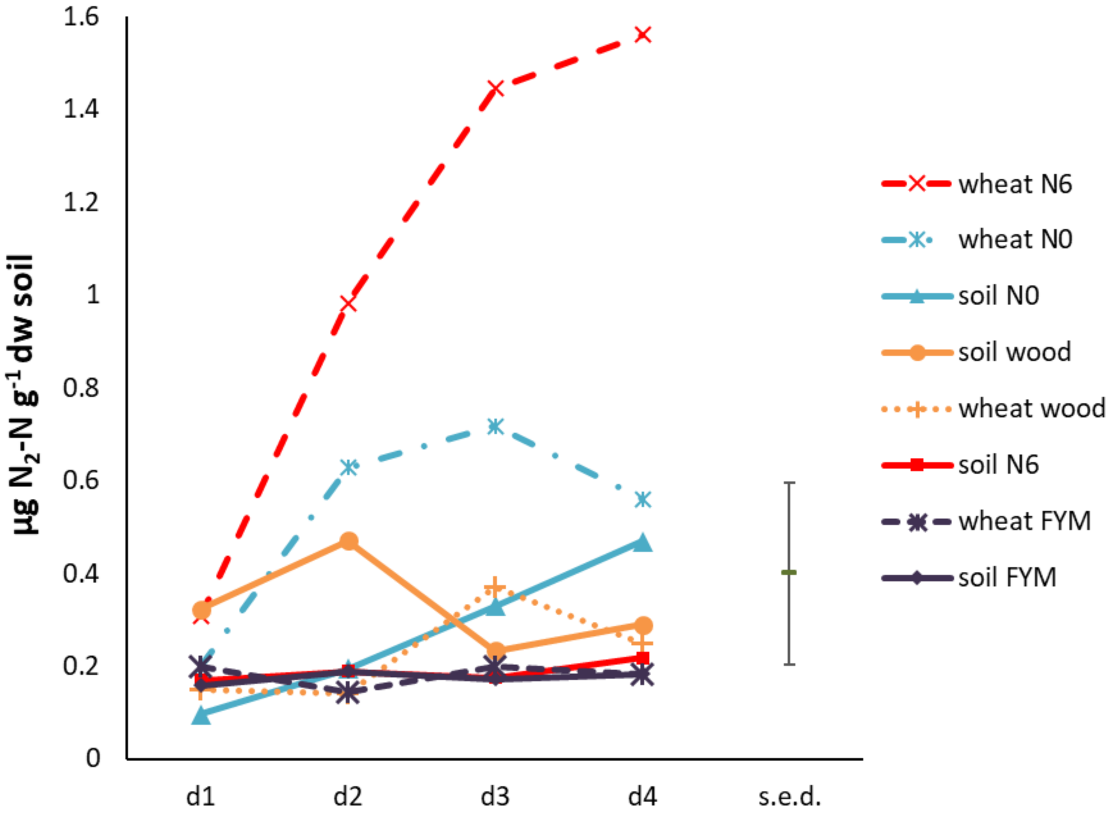

Supplementary Table S1. ANOVA for denitrification gene ratios.

		Ratio gene copy number from qPCR	
	**d.f.**	***nirK:nirS***	***nir:nos***
**soil**	$F_{3, 32}$	70.94, P <.001	11.58, P <.001
**fertilizer**	$F_{1, 32}$	NS	NS
**plant**	$F_{1, 32}$	NS	NS
**soil x fertilizer**	$F_{3, 32}$	NS	NS
**soil x plant**	$F_{3, 32}$	NS	NS
**fertilizer x plant**	$F_{1,32}$	NS	NS
**soil x plant x fertilizer**	$F_{3, 32}$	NS	NS

Supplementary Table S2a. Spearman's rank correlations for all 48 pots showing significant *r* values where *P* (no correlation) is < 0.05

	N_2O-N	CO_2	wfps	NO_3-N	NH_4-N	*16S*	*nirK*	*nirS*	*nosZ*I	*nosZ*II	*nir:nos*
CO_2	0.28										
wfps	0.42	0.04									
NO_3-N	0.61	0.58	0.43								
NH_4-N	-0.14	0.24	-0.40	-0.07							
16S	0.04	0.38	-0.05	0.16	0.70						
nirK	0.06	0.42	-0.14	0.22	0.72	0.87					
nirS	0.13	-0.25	0.38	0.06	0.07	0.34	0.26				
*nosZ*I	0.17	0.36	0.14	0.28	0.66	0.91	0.89	0.49			
*nosZ*II	0.12	0.27	0.15	0.21	0.60	0.93	0.79	0.59	0.92		
nir:nos	-0.17	0.34	-0.50	0.02	0.53	0.38	0.65	-0.27	0.27	0.21	
nirK:nirS	-0.03	0.45	-0.36	0.13	0.61	0.59	0.81	-0.23	0.54	0.41	0.87

Supplementary Table S2b. Spearman's rank correlations for 24 pots where $K^{15}NO_3$ was applied showing significant *r* values where *P* (no correlation) is < 0.05

	N_2O-N	N_2-N	total N	N_2O%	^{15}Natom%	CO_2	wfps	NO_3-N	NH_4-N	*16S*	*nirK*	*nirS*	*nosZ*I	*nosZ*II	*nir:nos*
N_2-N	0.47														
total N	0.54	0.99													
N_2O%	0.87	0.13	0.19												
^{15}Natom%	0.87	0.44	0.50	0.66											
CO_2	-0.24	-0.17	-0.18	-0.08	-0.48										
wfps	0.46	0.07	0.06	0.56	0.25	-0.14									
NO_3-N	0.01	-0.28	-0.30	0.30	-0.29	0.40	0.54								
NH_4-N	-0.39	-0.20	-0.22	-0.45	-0.49	0.30	-0.46	-0.21							
16S	-0.21	-0.22	-0.22	-0.13	-0.48	0.47	0.02	0.26	0.60						
nirK	-0.22	-0.15	-0.16	-0.23	-0.48	0.45	-0.03	0.17	0.66	0.90					
nirS	0.25	-0.20	-0.19	0.27	0.25	-0.27	0.41	0.16	-0.04	0.30	0.27				
*nosZ*I	-0.10	-0.18	-0.19	-0.08	-0.36	0.35	0.20	0.22	0.57	0.88	0.89	0.51			
*nosZ*II	-0.09	-0.27	-0.27	-0.01	-0.30	0.30	0.20	0.33	0.48	0.91	0.81	0.60	0.90		
nir:nos	-0.41	-0.15	-0.15	-0.44	-0.51	0.39	-0.51	0.03	0.64	0.51	0.65	-0.24	0.32	0.35	
nirK:nirS	-0.39	-0.13	-0.13	-0.38	-0.58	0.50	-0.39	0.01	0.67	0.66	0.78	-0.26	0.54	0.44	0.87

Total N = total gaseous N measured, i.e. N_2O-N + N_2-N; N_2O% = N_2O/total N; ^{15}N atom% is %^{15}N measured in N_2O-N. Mean values for gasses over 4 d sampling period were used; values that are related by co-derivation e.g. total N and N_2-N or N_2O-N are expected to correlate and are not commented on in the text.

Supplementary Table S3. Primers used for qPCR to assess denitrification gene abundance and activity

gene	primer	sequence	reference
**Bacterial 16SrRNA**	341F	CCT AYG GGR BGC ASC AG	Glaring et al., 2015[1]
	806R	GGA CTA CNN GGG TAT CTA AT	
nirK	nirK876F	ATY GGC GGV CAY GGC GA	Henry et al., 2004[2]
	nirK1040R	GCC TCG ATC AGR TTR TGG TT	Hallin and Lindgren, 1999[3]
nirS	cd3a F	GTS AAC GTS AAG GAR ACS GG	Michotey et al., 2000[4]
	R3cd R	GAS TTC GGR TGS GTC TTG A	Hallin and Lindgren, 1999[3]
***nosZ* Clade I**	nosZ2F	CGC RAC GGC AAS AAG GTS MSS GT	Henry et al., 2006[5]
	nosZ2R	CAK RTG CAK SGC RTG GCA GAA	
***nosZ* Clade II**	nosZIIF_1162-1178	CTI GGI CCI YTK CAY AC	Jones et al., 2013[6]
	nosZIIR_1889 -1907	GCI GAR CAR AAI TCB GTR C	

Supplementary Table S4. PCR primer efficiency in qPCR reactions

	gene target	efficiency (%)	r^2	slope	y-int
**DNA**	16S rRNA Bacteria	84.7	0.999	-3.751	35.845
	nirK	89	0.999	-3.618	35.832
	nirS	86.1	0.999	-3.708	33.375
	*nosZ*I	90.5	0.997	-3.572	35.427
	*nosZ*II	86.4	0.999	-3.697	32.52
**RNA**	16S rRNA Bacteria	82.8	0.996	-3.815	36.822
	nirK	87	0.989	-3.677	35.871
	nirS	83.8	0.999	-3.784	34.201
	*nosZ*I	82.9	0.998	-3.813	35.231
	*nosZ*II	87.5	0.998	-3.664	33.597

References

1 Glaring, M. A. *et al.* Microbial Diversity in a Permanently Cold and Alkaline Environment in Greenland. *Plos One* **10**, doi:10.1371/journal.pone.0124863 (2015).

2 Henry, S. *et al.* Quantification of denitrifying bacteria in soils by nirK gene targeted real-time PCR. *J. Microbiol. Methods* **59**, 327-335, doi:10.1016/j.mimet.2004.07.002 (2004).

3 Hallin, S. & Lindgren, P. E. PCR detection of genes encoding nitrile reductase in denitrifying bacteria. *Appl. Environ. Microbiol.* **65**, 1652-1657 (1999).

4 Michotey, V., Mejean, V. & Bonin, P. Comparison of methods for quantification of cytochrome cd(1)-denitrifying bacteria in environmental marine samples. *Appl. Environ. Microbiol.* **66**, 1564-1571, doi:10.1128/aem.66.4.1564-1571.2000 (2000).

5 Henry, S., Bru, D., Stres, B., Hallet, S. & Philippot, L. Quantitative detection of the nosZ gene, encoding nitrous oxide reductase, and comparison of the abundances of 16S rRNA, narG, nirK, and nosZ genes in soils. *Appl. Environ. Microbiol.* **72**, 5181-5189, doi:10.1128/AEM.00231-06 (2006).

6 Jones, C. M., Graf, D. R. H., Bru, D., Philippot, L. & Hallin, S. The unaccounted yet abundant nitrous oxide-reducing microbial community: a potential nitrous oxide sink. *Isme Journal* **7**, 417-426, doi:10.1038/ismej.2012.125 (2013).
